# Psychological strain among Chinese master’s students: protective mechanisms of self-esteem and influences of urban–rural background and gender

**DOI:** 10.3389/fpsyg.2026.1729754

**Published:** 2026-02-20

**Authors:** Wei Li, Enshan Cui

**Affiliations:** 1 School of Marxism, Harbin Normal University, Harbin, China; 2 Jiamusi University, Jiamusi, China; 3 University of International Business and Economics, Beijing, China; 4 Renmin University of China, Beijing, China

**Keywords:** Chinese master’s students, collaborative education, gender differences, psychological strain, rural–urban disparity, self-esteem

## Abstract

**Introduction:**

In China, master’s students form a large and growing population confronting unique developmental challenges, such as academic pressure and career uncertainty. However, specialized mental health research targeting this group, particularly focusing on the interplay between demographic factors and psychological resources, remains underdeveloped. This study investigates how residential background (rural vs. urban) and gender predict psychological strain among Chinese master’s students, as well as the predictive and moderating roles of self-esteem in this relationship.

**Methods:**

A cross-sectional survey was conducted in August 2025 among 315 master’s students at Jiamusi University, a public university in northeastern China. Participants completed the Psychological Strain Scale (PSS; *α* = 0.96) and the Rosenberg Self-Esteem Scale (RSES; *α* = 0.82). Data were analyzed using Pearson correlations, hierarchical regression, independent samples t-tests.

**Results:**

Rural students reported significantly higher total psychological strain and higher scores on all strain dimensions (aspiration, coping, deprivation, value strains) than their urban peers, with the largest difference observed in deprivation strain (Cohen’s *d* = 0.59). Males exhibited significantly higher deprivation strain than females (*d* = 0.29). Self-esteem emerged as the strongest negative predictor of total strain (*β* = −0.66, *p* < 0.001) and all dimension-specific strains. Furthermore, the protective effect of self-esteem was significantly stronger for females than for males (interaction *B* = −0.65, SE = 0.23, *t* = −2.83, *p* = 0.004), whereas its effect was consistent across rural and urban groups.

**Discussion:**

Psychological strain in Chinese master’s students is shaped by the complex interplay of structural (rural–urban background), cultural (gender roles), and psychological (self-esteem) factors. Self-esteem serves as a universal protective asset. Interventions should integrate structural equity measures, gender-responsive support, and universal self-esteem cultivation. Establishing a university-family-society collaborative education mechanism is crucial for effective mental health promotion.

## 1 Introduction

### 1.1 Research background

According to *The National Report on the Development of National Mental Health in China (2023–2024)*, the mental health status of adults is closely related to gender and urban–rural background. Notably, young people are a high-risk group for mental health issues, and within this group, the depression level of those aged 18–24 reaches its peak, a range that largely covers Chinese master’s students. Currently, there is abundant research on the mental health of undergraduate students, and studies on doctoral students have also been conducted. For example, a 2017 study found that the incidence of anxiety, depression, and burnout among doctoral students was higher than that among undergraduates and the general population (Levecque et al., 2017). According to statistics from the Ministry of Education of the People’s Republic of China, the number of on-campus master’s students in China had reached 3.4192 million by 2024. Against the backdrop of such a large population, their mental health status deserves full attention. However, compared with undergraduates and doctoral students, specialized mental health research on master’s students remains relatively scarce. Emerging research has begun to address this gap, highlighting trends such as the complex, non-linear evolution of mental health among Chinese university students (Hang et al., 2026), and identifying specific risk and protective mechanisms for graduates, including the pathways from stress to depression (Li et al., 2025) and the roles of factors like psychological flexibility (Wang et al., 2025). Despite this progress, a critical and underexplored area persists: the function of self-esteem as a core psychological resource in mitigating multidimensional psychological strain among master’s students, and whether its protective effect varies systematically across fundamental socio-structural divides, namely urban–rural background and gender. This gap limits the development of tailored support strategies. Therefore, a systematic analysis of the multidimensional composition of psychological strain among master’s students and its protective mechanisms is not only theoretically necessary but also practically urgent.

### 1.2 Psychological strain and self-esteem

In the field of mental health research, the dynamic interaction between psychological strain and self-esteem is a key concept for understanding individual adaptation and development. Based on the Suicide Strain Theory (STS), psychological strain is not a unidimensional feeling; it specifically manifests in four dimensions: Aspiration Strain (pressure from unmet academic or career expectations), Coping Strain (difficulty in managing academic tasks or life challenges), Deprivation Strain (a sense of loss due to insufficient resources or opportunities), and Value Strain (conflict between personal values and external norms) (Zhang and Lester, 2008). These dimension-specific strains collectively constitute the main psychological challenges faced by master’s students. In contrast, self-esteem, defined as an individual’s overall evaluation of self-worth (Rosenberg, 1965), plays a critical role in coping with strain. This internal self-evaluation system is the cornerstone of individual development, and it is essential for individuals to cope with daily strain and promote psychological well-being (Orth and Robins, 2014).

A large body of research has shown that high self-esteem is closely associated with positive psychophysiological states (Tsaousis, 2016) and enhances individuals’ resilience against negative impacts (Trzesniewski et al., 2003). In contrast, low self-esteem is significantly correlated with increased anxiety and adaptive difficulties (Pyszczynski et al., 2004). Longitudinal studies further confirm that self-esteem is a key predictor of subsequent depression and life satisfaction (Orth and Robins, 2014). However, the pathways through which self-esteem influences mental health, especially its protective and moderating mechanisms under specific strain dimensions and different demographic backgrounds, remain to be further explored.

### 1.3 Research rationale

This study focuses on mental health issues among master’s students. This group is in a critical transitional phase from undergraduate education to doctoral studies or professional development, confronting unique challenges including academic and research pressures, career uncertainty, and in-depth self-identity exploration (Han and Wang, 2024). These complex stressors collectively constitute a mental health issue that warrants in-depth investigation.

The selection of self-esteem as the core variable is based on its crucial role in the stress-coping process. Research indicates that self-esteem profoundly influences students’ perception patterns and coping strategies when dealing with stressors (Zhang et al., 2024) Within the specific context of Chinese higher education, the protective effect of self-esteem is likely moderated by structural factors. Specifically, resource disparities stemming from urban–rural background (Wan et al., 2025; Zheng and Postiglione, 2025) and gender role expectations (Xin et al., 2024) may interact complexly with self-esteem, a dynamic that collectively shapes students’ mental health trajectories.

Current research is constrained by two primary limitations. First, the prevalent use of single-indicator or composite measures often fails to adequately capture the multidimensional structure of psychological strain. Psychological strain is a complex construct comprising distinct dimensions such as aspiration, coping, deprivation, and value strains (Zhang et al., 2020). Relying solely on a single or composite score obscures the unique mechanisms and antecedents associated with each dimension, thereby oversimplifying our understanding of how strain develops and functions (Crosswell and Lockwood, 2020). This methodological constraint further impedes the development of targeted intervention strategies (Hasson et al., 2023). Second, research on the boundary conditions of the protective effect of self-esteem, particularly the moderating role of key socio-demographic variables, remains insufficient. Substantial evidence supports the role of self-esteem as a crucial resource buffering against mental health risks (Cui et al., 2024). Concurrently, urban–rural background and gender are established as significant variables contributing to differential experiences of strain: the urban–rural divide is closely linked to mental health disparities (Yu et al., 2022), and gender-specific patterns of strain shaped by role conflict have been consistently validated (Daoultzis and Kordoutis, 2024). However, how urban–rural background and gender moderate the protective effect of self-esteem on psychological strain has not yet been systematically examined within a unified analytical framework. Within the socio-cultural context of China, which emphasizes collective achievement and family responsibilities (Liu et al., 2019) and exhibits pronounced urban–rural disparities (Tuersun et al., 2025), a thorough investigation of this moderating mechanism holds significant theoretical value and practical implications for developing a stratified psychological support system tailored to the local context. Based on this, this study constructs an integrated theoretical framework to systematically examine the protective effect of self-esteem on mental health, as well as the moderating effects of urban–rural background and gender factors on this protective mechanism. The aim is to provide empirical evidence for developing targeted psychological intervention programs tailored to different subgroups of master’s students.

### 1.4 Theoretical framework

This study integrates three complementary theoretical perspectives, spanning macro-structural to micro-psychological levels, to construct a multi-layered explanatory framework. Guided by the principles of multilevel theory building (Klein et al., 1999), we establish an integrated analytical framework that achieves organic connection across theoretical dimensions. In the current study, gender (female/male) is used as a proxy to investigate outcomes that may be influenced by such social role dynamics.

From the macro-structural perspective, Bourdieu’s Theory of Capital and Field (Bourdieu, 2002) reveals the structural origins of urban–rural disparities in psychological strain. Seminal work in this field demonstrates how the unequal distribution of economic, cultural, and social capital translates into structural oppression through “symbolic violence.” This oppression manifests as internalized anxiety about social status, a dynamic that systematically increases psychological strain among students from rural backgrounds.

From the meso-role perspective, Eagly’s Social Role Theory (Eagly, 1987) elucidates gender-differentiated patterns in strain experiences. The cultural norms of “agency” (male-oriented traits such as achievement and dominance) and “communion” (female-oriented traits such as care and cooperation) shape distinct vulnerability profiles: male students show heightened sensitivity to deprivation strain due to the achievement pressures inherent in “agentic” roles, while female students navigate complex conflicts between “communal” expectations and academic aspirations.

From the micro-motivational perspective, Self-Determination Theory (Deci and Ryan, 2000) identifies self-esteem as a core protective mechanism. As an indicator of the satisfaction of the basic psychological need for “competence,” self-esteem enables individuals to reframe environmental challenges as manageable tasks rather than threats to self-worth, a function that serves as a crucial buffer in the stressor-strain relationship.

Bourdieu’s theory explains how urban–rural capital disparities—operating at the macro-structural level—shape strain; Eagly’s social role theory clarifies how gender norms, which function at the meso-role level, moderate strain vulnerability; and Self-Determination Theory reveals how self-esteem, acting as a micro-psychological resource, buffers strain. Together, they form a chain: structural factors influence role expectations, which in turn interact with individual resources to affect psychological strain.

Through this multilevel framework, the study captures the dynamic interactions between social structure (urban–rural background), cultural roles (gender), and personal resources (self-esteem). This integrated approach transcends the limitations of both structural determinism and decontextualized individual psychology, providing a robust foundation for hypothesis development.

### 1.5 Research hypotheses

Based on the above theoretical framework and literature review, the following research hypotheses are proposed:

*H1*: Rural master’s students will report significantly higher overall psychological strain and higher scores on all dimension-specific strains than urban master’s students.

*H2*: Male master’s students will report higher Deprivation Strain than female master’s students, and the protective effect of self-esteem on strain will be stronger for female master’s students.

*H3*: Self-esteem will be negatively correlated with overall psychological strain and all dimension-specific strains, and this correlation will be consistent across urban and rural master’s student groups.

### 1.6 Research objectives

To test the above hypotheses, this study aims to: (1) explore how urban–rural background and gender predict overall psychological strain and dimension-specific strains among master’s students; (2) examine the predictive role of self-esteem and its moderating role in the relationship between demographic variables and strain; (3) quantify and verify strain differences related to urban–rural background and gender using effect sizes; and (4) propose integrated, multi-level intervention recommendations based on empirical results.

## 2 Methods

### 2.1 Study design and participants

A cross-sectional design was adopted in this study. Data were collected at Jiamusi University, a public comprehensive university in Northeast China, via the online questionnaire platform Wenjuanxing. This study was reviewed and approved by the Biomedical Ethics Committee of Jiamusi University (Approval No. JMSU-2025080101). A total of 315 valid questionnaires were retrieved. The demographic characteristics of the sample were as follows: 204 females (64.76%) and 111 males (35.24%); in terms of urban–rural background, 125 participants were from rural areas (39.68%) and 190 from urban areas (60.32%). Before participating in the survey, all participants were clearly informed of the voluntary nature of the study and were told that data would be strictly processed anonymously and used only for academic purposes. Completion and submission of the questionnaire were considered as informed consent.

### 2.2 Measures

Two reliable measurement tools, validated in Chinese student populations, were used in this study. All instruments were administered in Chinese.

The Chinese version of the 20-item Psychological Strain Scale (PSS) (Zhang, 2016) was used to assess participants’ overall psychological strain and scores on four dimension-specific strains (5 items per dimension: Aspiration Strain, Coping Strain, Deprivation Strain, and Value Strain). A 4-point Likert scale was used (1 = Strongly Disagree to 4 = Strongly Agree). Total scores ranged from 20 to 80, and subscale scores ranged from 5 to 20; higher scores indicated higher levels of strain. In this study, the Cronbach’s *α* coefficients of the scale were excellent: α = 0.96 for the total scale, 0.86 for Aspiration Strain, 0.92 for Coping Strain, 0.92 for Deprivation Strain, and 0.93 for Value Strain.

The Chinese version of the 10-item Rosenberg Self-Esteem Scale (RSES) (Jiang et al., 2023; Rosenberg, 1965) was used to assess overall self-esteem. A 4-point Likert scale was used (1 = Strongly Disagree to 4 = Strongly Agree), with 5 items scored in reverse. Total scores ranged from 10 to 40; higher scores indicated higher levels of self-esteem. In this study, the Cronbach’s α coefficient of the scale was 0.82, demonstrating good internal consistency.

### 2.3 Statistical analysis

All data were analyzed using SPSS 26.0. Continuous variables were described using mean (*M*) and standard deviation (*SD*); categorical variables were described using frequency (*n*) and percentage (%). Pearson correlation analysis was used to test the bivariate relationships between self-esteem, demographic variables, and overall/dimension-specific strains. Hierarchical regression analysis was conducted in two steps to predict overall psychological strain: Step 1 included only demographic variables (gender, urban–rural background), and Step 2 added self-esteem. The incremental validity of self-esteem was quantified using the coefficient of determination increment (Δ*R*^2^).

Separate hierarchical regression analyses were conducted to test the moderating roles of gender and urban–rural background. For significant interaction effects, simple slope analysis was performed to decompose the nature of the moderation. In all analyses, self-esteem served as the independent variable, overall psychological strain as the dependent variable, and either gender or urban–rural background as the moderator. For these moderation analyses, to reduce multicollinearity and facilitate the interpretation of interaction effects, the continuous predictor (self-esteem) and the outcome variable (psychological strain) were mean-centered prior to creating the interaction term(s). In contrast, the original (uncentered) variables were used for the Pearson correlation analyses and for the initial hierarchical regression models that did not include interaction terms. To ensure the robustness of parameter estimates, all analyses employed the Bootstrapping method (with 5,000 resamples) to calculate standard errors and confidence intervals.

The same two-step hierarchical regression model (Step 1: demographic variables; Step 2: adding self-esteem) was applied to predict each dimension-specific strain (e.g., aspiration strain, coping strain), following the same logic used for overall psychological strain. Independent samples *t*-tests were used to compare strain scores between urban and rural groups, and between male and female groups, with effect sizes represented by Cohen’s *d* (small effect: *d* = 0.2; medium effect: *d* = 0.5; large effect: *d* = 0.8) (Cohen, 1988).

All tests were two-tailed, with a significance level set at *p* < 0.05. It is important to note that the cross-sectional design of this study limits the ability to draw causal inferences. The reported predictive and moderating relationships should be interpreted as observed associations, which do not establish causality.

## 3 Results

The following analyses are based on cross-sectional data, and the results primarily reflect associations among the variables. Causal interpretations require future longitudinal or experimental verification.

### 3.1 Descriptive statistics and correlation analysis

As shown in Table 1, the overall psychological strain score of the sample was *M* = 41.23 (*SD* = 11.58). Among the four dimension-specific strains, Aspiration Strain had the highest score (*M* = 10.97, *SD* = 3.52), while Coping Strain had the lowest score (*M* = 9.57, *SD* = 3.14). The self-esteem score of the master’s students (*M* = 30.77, *SD* = 4.28) was similar to the results of previous surveys on Chinese master’s student populations (Cui et al., 2024).

**Table 1:** Descriptive statistics and Pearson correlations of key variables (*N* = 315).

Variable	M	SD	1	2	3	4	5	6
1. Self-esteem	30.77	4.28	—					
2. Total psychological strain	41.23	11.58	−0.68**	—				
3. Coping strain	9.57	3.14	−0.63**	0.90**	—			
4. Deprivation strain	10.17	3.14	−0.57**	0.88**	0.75**	—		
5. Aspiration strain	10.97	3.52	−0.57**	0.90**	0.73**	0.74**	—	
6. Value strain	10.52	3.24	−0.63**	0.87**	0.74**	0.63**	0.72**	—

Urban–Rural Background: 0, Rural; 1, Urban. Gender: 0, Female; 1, Male. *p < 0.05, **p < 0.01 (two-tailed).

Correlation analysis showed that overall psychological strain was strongly positively correlated with all four dimension-specific strains (*r* = 0.87–0.90, all *p* < 0.01). Self-esteem was significantly negatively correlated with overall psychological strain (*r* = −0.68, *p* < 0.01) and was also negatively correlated with each dimension-specific strain (Aspiration Strain: *r* = −0.57; Coping Strain: *r* = −0.63; Deprivation Strain: *r* = −0.57; Value Strain: *r* = −0.63; all *p* < 0.01). Group differences in strain based on gender and urban–rural background were analyzed using independent samples *t*-tests and are reported in Section 3.4.

### 3.2 Hierarchical regression analyses: overall and dimension-specific psychological strain

Table 2 integrates the hierarchical regression results for overall psychological strain and the four dimension-specific strains. For all strain variables, the variance explained by the model increased significantly after adding self-esteem in Step 2, confirming the universal predictive role of self-esteem.

**Table 2:** Hierarchical regression analysis of overall and dimension-specific psychological strain (*N* = 315).

Dependent variable	Step 1: demographic predictors (*β*)		Step 2: + self-esteem				Model fit indices		
	Urban–rural background	Gender	Urban–rural background (*β*)	Gender (*β*)	Self-esteem (*β*)	Self-esteem (B [95% CI])	*R*^2^ (Step 1)	Δ*R*^2^ (Step 2)	ΔF (Step 2)
Overall psychological strain	−0.23***	0.05	−0.10*	−0.01	−0.66***	−1.78 [−2.00, −1.55]	0.06	0.41***	239.86***
Coping strain	−0.17***	0.04	−0.04	−0.02	−0.63***	−0.46 [−0.52, −0.40]	0.03	0.37***	195.20***
Deprivation strain	−0.28***	0.12*	−0.17***	0.07	−0.53***	−0.39 [−0.46, −0.32]	0.09	0.27***	129.99***
Aspiration strain	−0.19***	0.00	−0.08	−0.05	−0.56***	−0.46 [−0.54, −0.38]	0.04	0.30***	140.32***
Value strain	−0.18***	0.03	−0.05	−0.02	−0.62***	−0.47 [−0.54, −0.40]	0.03	0.37***	188.73***

Urban–Rural Background: 0, Rural; 1, Urban. Gender: 0, Female; 1, Male. *p < 0.05, **p < 0.01, ***p < 0.001. Δ*R*^2^, Incremental variance explained by adding self-esteem; ΔF, F-statistic for Δ*R*^2^; the confidence intervals (CI) for the unstandardized coefficients (B) are bias-corrected bootstrap confidence intervals based on 5,000 resamples.

For overall psychological strain, Step 1 explained 6% of the variance (*R*^2^ = 0.06, *F* = 9.37, *p* < 0.001), with urban–rural background as the only significant predictor (*β* = −0.23, *p* < 0.001). In Step 2, after adding self-esteem, the variance explained by the model increased significantly to 47% (Δ*R*^2^ = 0.41, Δ*F* = 239.86, *p* < 0.001), and self-esteem became the strongest negative predictor (*β* = −0.66, *p* < 0.001). After controlling for the effect of self-esteem, urban–rural background remained a significant negative predictor (*β* = −0.10, *p* < 0.05), while the predictive effect of gender remained non-significant (*β* = −0.01, *p* = 0.82).

For Coping Strain, Step 1 explained 3% of the variance (*R*^2^ = 0.03, *F* = 5.00, *p* < 0.01), with urban–rural background as a significant predictor (*β* = −0.17, *p* < 0.01). Step 2 increased the explained variance to 40% (Δ*R*^2^ = 0.37, Δ*F* = 195.20, *p* < 0.001), and self-esteem was the key predictor (*β* = −0.63, *p* < 0.001). The effect of urban–rural background became non-significant in Step 2 (*β* = −0.04, *p* = 0.32).

For Deprivation Strain, Step 1 explained 9% of the variance (*R*^2^ = 0.09, *F* = 16.15, *p* < 0.001), with both urban–rural background (*β* = −0.28, *p* < 0.001) and gender (*β* = 0.12, *p* < 0.05) as significant predictors. Step 2 increased the explained variance to 36% (Δ*R*^2^ = 0.27, Δ*F* = 129.99, *p* **<** 0.001), and self-esteem significantly predicted Deprivation Strain (*β* = −0.53, *p* < 0.001). In Step 2, urban–rural background remained significant (*β* = −0.17, *p* < 0.001) while gender became non-significant (*β* = 0.07, *p* = 0.13).

For Aspiration Strain, Step 1 explained 4% of the variance (*R*^2^ = 0.04, *F* = 6.14, *p* < 0.01), with urban–rural background as the only significant predictor (*β* = −0.19, *p* < 0.001). Step 2 increased the explained variance to 34% (Δ*R*^2^ = 0.30, Δ*F* = 140.32, *p* < 0.001), and self-esteem became the strongest predictor (β = −0.56, *p* < 0.001). The effect of urban–rural background became non-significant in Step 2 (*β* = −0.08, *p* = 0.09).

For Value Strain, Step 1 explained 3% of the variance (*R*^2^ = 0.03, *F* = 5.35, *p* < 0.01), with urban–rural background as a significant predictor (*β* = −0.18, *p* < 0.01). Step 2 increased the explained variance to 40% (Δ*R*^2^ = 0.37, Δ*F* = 188.73, *p* < 0.001), and self-esteem became the strongest predictor (*β* = −0.62, *p* < 0.001). The effect of urban–rural background became non-significant in Step 2 (*β* = −0.05, *p* = 0.24).

### 3.3 Moderation analysis: roles of gender and urban–rural background

To examine the moderating roles of gender and urban–rural background in the relationship between self-esteem and overall psychological strain, two separate hierarchical regression analyses were conducted. All continuous variables were mean-centered prior to analysis. To ensure the robustness of parameter estimates, all analyses employed the Bootstrapping method (with 5,000 resamples) to calculate standard errors and confidence intervals.

**Figure 1:**
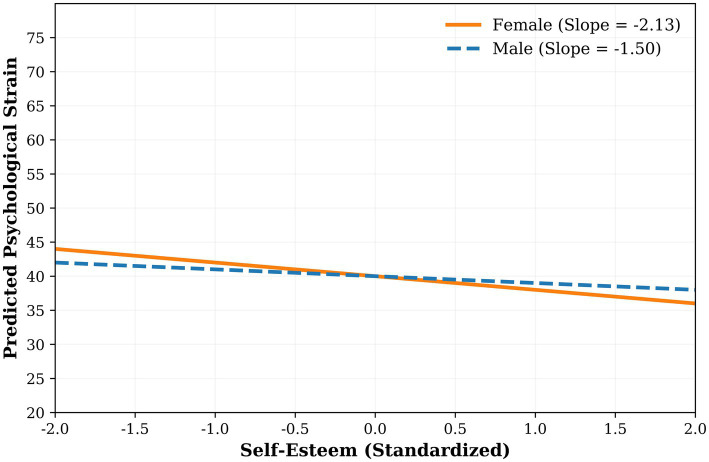
Interaction plot of self-esteem × gender on overall psychological strain. Alt text: Interaction plot with standardized self-esteem (*x*-axis, range: −2.0 to 2.0) and overall psychological strain (*y*-axis, range: 20 to 80) stratified by gender. Female students (slope = −2.13, *p* < 0.001) show a steeper negative slope than male students (slope = −1.50, *p* < 0.001), indicating a stronger protective effect of self-esteem on psychological strain in females. Gender: 0 = Female, 1 = Male. Self-esteem levels are mean-centered (Low, Mean − 1 *SD*; Mean, Average; High, Mean + 1 *SD*). The steeper slope for females indicates a stronger protective effect of self-esteem on psychological strain.

First, the moderating role of gender was tested. After controlling for the main effect of self-esteem, gender and the interaction term between self-esteem and gender were entered into the regression model sequentially. The results (Table 3, Panel A) showed that the interaction effect (self-esteem × gender) was significant (*B* = −0.65, *SE* = 0.23, *t* = −2.83, *p* = 0.005, Bootstrap 95% *CI* [−1.11, −0.20]), explaining an additional 1% of the variance in strain (Δ*R*^2^ = 0.01). To decompose this significant interaction, a simple slope analysis was performed. The results (presented in Table 4 and Figure 1) revealed that the negative predictive effect of self-esteem on psychological strain was significant for both female and male students, but was stronger for females (slope = −2.13, *p* < 0.001) and weaker for males (slope = −1.50, *p* < 0.001). This pattern, indicating a stronger buffering effect of self-esteem for females, is consistent with Hypothesis H2. It is worth noting that the main effect of gender on psychological strain was not significant in this moderation model (*B* = −0.36, *p* = 0.72).

**Table 3:** Moderation analysis of gender and urban–rural background (*N* = 315).

Model and predictors	B	SE	*t*	*p*	95% CI	Δ*R*^2^
A. Gender moderation model	–	–	–	–	–	–
Constant	41.10	0.48	85.85	< 0.001***	[40.16, 42.04]	—
Self-esteem	−1.82	0.11	−16.21	<0 0.001***	[−2.04, −1.60]	—
Gender	−0.36	1.00	−0.36	0.72	[−2.34, 1.61]	—
Self-Esteem × Gender	−0.65	0.23	−2.83	0.005**	[−1.11, −0.20]	0.01
B. Urban–rural background moderation model	–	–	–	–	–	–
Constant	41.19	0.49	84.19	< 0.001***	[40.23, 42.15]	—
Self-esteem	−1.78	0.11	−15.52	<0 0.001***	[−2.00, −1.55]	—
Urban–rural background	−2.29	1.00	−2.27	0.02*	[−4.26, −0.31]	—
Self-esteem × Urban–rural background	0.09	0.24	0.36	0.72	[−0.38, 0.55]	0.00

Dependent Variable: Psychological Strain; Urban–Rural Background: 0, Rural; 1, Urban. Gender: 0, Female, 1, Male. All confidence intervals (95% CI) are bias-corrected bootstrap confidence intervals based on 5,000 resamples. B, unstandardized regression coefficient; SE, standard error. *p < 0.05, **p < 0.01, ***p < 0.001.

**Table 4:** Simple slope analysis for the significant self-esteem × gender interaction (*N* = 315).

Gender Level	Slope	SE	*t*	*p*	95% CI	
					Lower	Upper
Low (−1 SD: Female)	−2.13	0.15	−13.87	<0.001***	−2.43	−1.83
Mean (Average)	−1.82	0.11	−16.21	<0.001***	−2.04	−1.60
High (+1 SD: Male)	−1.50	0.16	−9.33	<0.001***	−1.82	−1.19

Dependent Variable: Overall Psychological Strain; Predictor: Self-Esteem; Gender: 0, Female; 1, Male; SD, Standard deviation. Simple slopes represent the change in psychological strain per 1-unit increase in self-esteem. ***p < 0.001.

Second, the moderating role of urban–rural background was tested. After controlling for the main effect of self-esteem, urban–rural background and the interaction term between self-esteem and urban–rural background were entered into the regression model sequentially. The results (Table 3, Panel B) indicated that the interaction effect (self-esteem × urban–rural background) was not significant (*B* = 0.09, *SE* = 0.24, *t* = 0.36, *p* = 0 0.72, Bootstrap 95% *CI* [−0.38, 0.55]), and did not increase the explanatory power of the model. This suggests that the protective effect of self-esteem showed no significant difference between rural and urban master’s students, confirming self-esteem as a universal protective factor independent of urban–rural background, which supports Hypothesis H3.

### 3.4 Group comparisons of psychological strain

Table 5 integrates the results of independent samples *t*-tests for gender and urban–rural groups, showing significant differences in strain scores between the groups. Regarding urban–rural background, rural master’s students reported significantly higher overall strain than urban master’s students (rural: *M* = 44.54 ± 10.73; urban: *M* = 39.04 ± 11.63; *t* = 4.23, *p* < 0.001, Cohen’s *d* = 0.48). Rural students also reported higher scores on all four dimension-specific strains: Coping Strain (rural: *M* = 10.24 ± 2.91; urban: *M* = 9.14 ± 3.22; *t* = 3.15, *p* < 0.01, *d* = 0.36), Deprivation Strain (rural: *M* = 11.26 ± 2.79; urban: *M* = 9.45 ± 3.16; *t* = 5.23, *p* < 0.001, *d* = 0.59), Aspiration Strain (rural: *M* = 11.81 ± 3.34; urban: *M* = 10.41 ± 3.53; *t* = 3.51, *p* < 0.001, *d* = 0.40), and Value Strain (rural: *M* = 11.23 ± 3.04; urban: *M* = 10.05 ± 3.28; *t* = 3.23, *p* < 0.01, *d* = 0.37). These results fully support Hypothesis H1.

**Table 5:** Group comparisons of psychological strain by gender and urban–rural background (*N* = 315; M ± SD, *t*, *p,* Cohen’s *d*).

Variable	Gender comparison (Female vs. Male)				Urban–rural comparison (Rural vs. Urban)			
	M ± SD	*t*	*p*	*d*	M ± SD	*t*	*p*	*d*
Self-esteem	31.07 ± 4.19 vs. 30.22 ± 4.42	1.69	0.09	0.20	29.69 ± 4.02 vs. 31.48 ± 4.32	−3.70	<0.001***	0.43
Overall psychological strain	40.75 ± 10.08 vs. 42.10 ± 13.94	−0.90	0.37	0.11	44.54 ± 10.73 vs. 39.04 ± 11.63	4.23	<0.001***	0.48
Coping strain	9.48 ± 2.70 vs. 9.76 ± 3.83	−0.69	0.49	0.08	10.24 ± 2.91 vs. 9.14 ± 3.22	3.15	0.002**	0.36
Deprivation strain	9.88 ± 2.76 vs. 10.69 ± 3.69	−2.03	0.04*	0.29	11.26 ± 2.79 vs. 9.45 ± 3.16	5.23	<0.001***	0.59
Aspiration strain	10.96 ± 3.21 vs. 10.97 ± 4.04	−0.03	0.98	0.00	11.81 ± 3.34 vs. 10.41 ± 3.53	3.51	<0.001***	0.40
Value strain	10.43 ± 2.90 vs. 10.68 ± 3.78	−0.59	0.55	0.08	11.23 ± 3.04 vs. 10.05 ± 3.28	3.23	0.001**	0.37

For group comparisons: Urban–Rural Background: 0, Rural; 1, Urban. Gender: 0, Female, 1, Male. Cohen’s *d* interpretation: 0.2, small effect; 0.5, medium effect; 0.8, large effect. *p < 0.05, **p < 0.01, ***p < 0.001.

In terms of gender differences, male master’s students reported significantly higher Deprivation Strain than female master’s students (male: *M* = 10.69 ± 3.69; female: *M* = 9.88 ± 2.76; *t* = 2.03, *p* = 0.04, *d* = 0.29). However, there were no significant gender differences in overall psychological strain (female: *M* = 40.75 ± 10.08; male: *M* = 42.10 ± 13.94; *t* = 0.90, *p* = 0.37) or the other three dimension-specific strains (all *p* > 0.05), which partially supports Hypothesis H2.

## 4 Discussion

This study systematically explored the complex effects of urban–rural background, gender, and self-esteem on overall psychological strain and dimension-specific strains among master’s students at Jiamusi University, China. By integrating Bourdieu’s Theory of Capital and Field, Eagly’s Gender Role Theory, and Deci and Ryan’s Self-Determination Theory, the results reveal the interactions between structural factors, cultural norms, and psychological resources in shaping master’s students’ mental health, providing a theoretical basis and practical direction for constructing targeted intervention systems.

### 4.1 Urban–rural differences: structural barriers and symbolic violence

This study found that rural master’s students reported significantly higher scores on overall strain and all dimension-specific strains than urban master’s students, with the most prominent difference observed in Deprivation Strain (Cohen’s *d* = 0.59, medium effect). This result strongly supports Bourdieu’s Theory of Capital and Field. Rural students face not only limitations in economic capital but also systemic disadvantages in cultural capital, such as differences in academic vision and discourse systems, and social capital, including mentor networks and academic connections. When rural students, carrying their original “habitus,” enter the academic field dominated by urban culture, this deep-seated mismatch is transformed into symbolic violence: their disadvantages are silently attributed to personal ability rather than structural injustice, a dynamic that systematically increases their psychological strain.

Notably, even after controlling for the effect of self-esteem, urban–rural background remained a significant predictor of overall strain (*β* = −0.10, *p* < 0.05), confirming the structural roots of strain differences between the two groups. This study rejects the misunderstanding that rural students’ strain is simply attributed to insufficient personal ability, and emphasizes that their strain experiences must be understood from the perspective of capital inequality and field adaptation. This finding provides key evidence for supporting the development of rural master’s students at the institutional level.

### 4.2 Gender differences: role expectations and differential vulnerabilities

Gender differences in psychological strain were only observed in the dimension of Deprivation Strain: male master’s students reported significantly higher scores than female master’s students (Cohen’s *d* = 0.29, small effect). This pattern of gender differences is consistent with the premises of Eagly’s Gender Role Theory, which may be related to traditional gender norms in China. Males are often assigned higher “agency” pressure of “achieving success and establishing a career,” making them more sensitive to resource deprivation, such as limited research opportunities or fewer awards, and peer comparisons in academic competition. Their academic achievements are deeply tied to their sense of self-worth, a connection that further amplifies the impact of deprivation experiences on psychological strain (Jin, 2023).

Meanwhile, the study found that the buffering effect of self-esteem on strain was stronger for female master’s students than for males (female slope = −2.13, male slope = −1.50, both *p* < 0.001). This result can be explained by the unique role conflicts faced by female master’s students: although females often outperform males in academic performance during higher education, they still face more barriers in obtaining senior academic positions, improving research performance, and advancing their careers in the long run (Cao et al., 2023). Additionally, females often need to balance academic aspirations with family role expectations, and external support for addressing such conflicts is usually insufficient. Therefore, females rely more on self-esteem, an internal psychological resource, to maintain psychological balance. This gendered pattern in coping reliance aligns with Eagly’s Social Role Theory. The theory suggests that the male “agentic” role encourages coping strategies oriented toward external achievement and status affirmation (Eagly, 1987). Empirical evidence supports this: men are more likely to engage in competitive behaviors and overt task participation as ways to assert competence and navigate challenges (Badura et al., 2018), a pattern that may reduce their reliance on internal self-evaluation for stress buffering. Furthermore, when masculine identity is threatened, men often exhibit responses aimed at restoring external perceptions of gender norm conformity, such as increased anger and compensatory self-presentation (Steiner et al., 2022), further underscoring an externally focused coping orientation. In contrast, males tend to adopt externally oriented coping strategies, such as focusing on competition and acquiring more achievements, a tendency that reduces their dependence on self-esteem resources. This finding reveals that gender differences in psychological strain are reflected not only in the types of strain experienced but also in the core psychological mechanisms used to cope with strain.

### 4.3 Self-esteem: a universal psychological asset

Self-esteem emerged as the strongest protective factor in this study, with a significant negative correlation with overall psychological strain (*r* = −0.68, *p* < 0.01) and all four dimension-specific strains (*r* = −0.57 to −0.63, all *p* < 0.01). In the hierarchical regression analysis, self-esteem explained 27 to 38% of the variance in each strain dimension (Δ*R*^2^ = 0.27–0.37, all *p* < 0.01), which fully aligns with the assertion of Self-Determination Theory. As a reflection of the fulfillment of the basic psychological need for “competence,” self-esteem helps individuals reframe academic challenges as manageable tasks rather than threats to self-worth, a function that effectively buffers the impact of stressors on psychological strain.

Crucially, the interaction effect of self-esteem × urban–rural background was not significant (*β* = 0.01, *p* = 0.72), meaning the protective effect of self-esteem showed no significant difference between rural master’s students (*β* = −0.67, *p* < 0.001) and urban master’s students (*B* = 0.09, *p* < 0.001). This result establishes self-esteem as a universal psychological asset for master’s students. It implies that interventions aimed at enhancing self-esteem can benefit all master’s students without distinction: for rural students, high self-esteem can enhance their sense of agency, motivating them to actively seek support and resources under conditions of capital constraints; for female students, high self-esteem can strengthen their self-identity, alleviating the psychological strain caused by conflicts between career development and role expectations.

### 4.4 Study limitations and future directions

This study has three limitations that need to be addressed in future research. First, the sample was drawn solely from a single public university (Jiamusi University) in Northeast China, which significantly limits the representativeness and generalizability of the findings. China is vast, with substantial variations in university tiers (e.g., “Double First-Class” vs. non-"Double First-Class”), institutional types (comprehensive, teacher-training, science and engineering, etc.), and regional economic development levels. These factors may all influence master’s students’ stress experiences and resource access. Therefore, conclusions drawn from this single-institution sample should be extrapolated to the broader population of “Chinese master’s students” with great caution. Future studies should adopt a multi-region and multi-type institutional sampling design to improve the generalizability of the findings to the broader Chinese master’s student population. Second, data were collected solely through self-report questionnaires, which may be subject to social desirability bias. Future studies can integrate multiple data sources, such as using semi-structured interviews to capture in-depth experiences and incorporating third-party evaluations for triangulation, a strategy that enhances the reliability and validity of research conclusions. Third, this study only tested the predictive and moderating roles of self-esteem, without exploring the potential mediating mechanisms through which urban–rural background, gender, and self-esteem influence psychological strain. Future research can construct “moderated mediation models” or “mediated moderation models” to fully reveal the complex pathways through which these factors affect psychological strain, deepening the understanding of the protective mechanism of self-esteem.

## 5 Conclusions and intervention implications

This study demonstrates that the psychological strain of master’s students at Jiamusi University is the result of complex interactions between macro-structural factors (urban–rural capital differences), meso-level role factors (gender norms), and micro-psychological factors (self-esteem). Bourdieu’s Theory of Capital and Field reveals the social roots of urban–rural differences in psychological strain; Eagly’s Gender Role Theory explains the gender-specific patterns of strain vulnerability; and Self-Determination Theory confirms the universal protective mechanism of self-esteem as an internal psychological resource. Therefore, it is crucial to construct an integrated intervention system that integrates “structural improvement, cultural adaptation, and psychological support” to address the psychological strain of master’s students; it should be noted that the conclusions are based on a single-institution sample, and caution is warranted when generalizing them to institutions of different regions or types.

### 5.1 Implement structural equity interventions to address resource gaps for rural master’s students

To address the systemic capital disadvantages faced by rural master’s students, universities should focus on both resource supply and field adaptation to promote structural equity. In terms of economic capital compensation, universities can open free access to high-quality academic databases, research equipment, and professional software, such as providing free licenses for statistical analysis tools and scientific computing software, to eliminate research barriers caused by economic limitations. For cultural capital cultivation, specialized courses on “academic norms and research methods” can be offered to help rural students quickly grasp the academic discourse system and research logic of higher education. Additionally, a “mentor-senior student” pairing system can be established, where senior urban students or outstanding rural alumni guide new rural students in academic writing, research project design, and academic communication, accelerating their adaptation to the academic field. In terms of social capital expansion, special research funds for rural master’s students can be set up to support their participation in national or regional academic conferences, fieldwork, and academic exchange activities. Meanwhile, a “rural master’s students-alumnus” connection platform can be built to help rural students expand their academic networks and obtain more career development resources.

### 5.2 Promote gender-responsive support to address differential strain vulnerabilities

Targeted intervention strategies should be designed based on the gender-specific patterns of psychological strain. To alleviate the higher Deprivation Strain experienced by male master’s students, universities can organize activities such as “academic setback sharing sessions,” where senior students or teachers share their experiences of overcoming academic failures (e.g., rejected papers, failed project applications), helping male students rationalize setbacks and reduce their over-reliance on “achievement-only” values. Additionally, “non-utilitarian research practice” activities, such as community service-oriented research or interdisciplinary exploratory projects, can be carried out to help males shift their focus from “resource competition” to “research interest” and “social value,” a shift that reduces the psychological pressure caused by resource deprivation. To strengthen the buffering effect of self-esteem for female master’s students, “female scholar development workshops” can be established, inviting outstanding female scholars from various fields to share their experiences of balancing academic careers and family roles, and providing training on coping skills for role conflicts, such as time management and boundary setting. Furthermore, universities can promote “diverse success perspectives” through campus publicity and curriculum cases, reducing the normative expectations that males “must achieve great success” and females “must balance family and career,” and creating a more inclusive campus culture that respects individual choices.

### 5.3 Integrate self-esteem cultivation as a universal strategy into student support systems

Given the universal protective effect of self-esteem, universities should integrate self-esteem cultivation into the daily training and support systems for master’s students. First, optimize the mentor feedback model: train mentors to provide “strength-based feedback” that focuses on students’ specific progress and strengths, such as acknowledging improvements in research design or writing skills, rather than only pointing out deficiencies or making general evaluations, a practice that enhances students’ sense of “competence” and self-worth. Second, offer specialized self-esteem enhancement courses: embed “self-cognition and value construction” modules into mandatory mental health courses for master’s students, and carry out group counseling activities focused on self-esteem, such as cognitive restructuring training to help students challenge negative self-evaluations and self-compassion exercises to reduce self-criticism. Third, expand channels for students to realize their self-worth: encourage master’s students to participate in non-academic activities such as student leadership training, voluntary services (e.g., tutoring primary and secondary school students, participating in community mental health promotion), and interdisciplinary innovation projects, helping them develop multiple sources of self-esteem beyond academic achievements and reducing the psychological pressure caused by over-reliance on academic performance.

### 5.4 Build a collaborative education mechanism to form a supportive synergy

Addressing the psychological strain of master’s students requires more than the efforts of universities alone; it is necessary to construct a “university-family-society” collaborative education mechanism to form a supportive synergy. This approach is firmly grounded in the Chinese cultural context, where familial bonds and interdependence remain strong throughout adulthood. Empirical research on Chinese postgraduate students confirms that family functioning serves as a significant contextual factor influencing their psychological adaptation and academic development (Zhou et al., 2025). For master’s students, who are within this developmental stage, family continues to be a key source of support. Empirical studies confirm that parental autonomy support is significantly associated with better mental health (e.g., higher life satisfaction, fewer emotional problems) among Chinese emerging adults, with self-esteem playing a mediating role (Ma et al., 2022). Most pertinently, longitudinal research on Chinese master’s students specifically has demonstrated that family support significantly moderates the impact of family environment on depressive symptoms, highlighting its active protective role in this population (Ren et al., 2024). Therefore, engaging families is not an infantilization of adult students but a strategic leveraging of a culturally salient and empirically validated support system. Consequently, at the university-family level, regular communication channels should be established, such as issuing “parent newsletters” every semester to introduce the academic characteristics and psychological challenges of the master’s stage, and holding “online parent meetings” to share students’ academic progress and mental health status, helping families understand the actual situation of master’s students and guiding them to establish reasonable achievement expectations, a step that avoids the transmission of excessive family pressure to students. At the university-society level, universities can cooperate with local governments and enterprises to provide localized internship and employment support for rural master’s students, such as establishing off-campus practice bases in rural areas or providing policy preferences for rural students to return to their hometowns for employment, and connect female master’s students with “career development mentors” in enterprises and research institutions to provide professional guidance on career planning and role adaptation. At the on-campus resource integration level, a “mentor-counselor-mental health teacher” linkage mechanism should be established: mentors are responsible for identifying and addressing students’ academic strain, counselors track students’ daily psychological status and provide preliminary psychological support, and professional mental health teachers conduct in-depth interventions for students with severe strain or mental health risks, forming a comprehensive and multi-level psychological support network.

Alleviating the psychological strain of master’s students and safeguarding their mental health is a long-term and systematic project that requires the collaborative efforts of universities, families, society, and individuals. Only by integrating structural support to reduce environmental barriers, gender-responsive strategies to address specific vulnerabilities, universal psychological cultivation to enhance internal resources, and collaborative mechanisms to form support synergy can we build a more inclusive, supportive, and developmental educational environment, allowing every master’s student, regardless of their urban–rural background or gender, to achieve comprehensive development in both academic achievements and psychological well-being.

## Data Availability

The raw data supporting the conclusions of this article will be made available by the authors, without undue reservation.
